# Supplementary material for: Unicellular and Multicellular Modes of Selection Impose Distinct Constraints on Cellular Phenotype Evolution
Source: bioRxiv. 2026 Sep 17:2026.09.16.751889. Preprint. [Version 1] doi: 10.64898/2026.09.16.751889 (PMC13596391; doi:10.64898/2026.09.16.751889)
Supplement: Supplementary file 1 [file NIHPP2026.09.16.751889v1-supplement-1.pdf]

bioRxiv preprint doi: https://doi.org/10.64898/2026.09.16.751889; this version posted September 17, 2026. The copyright holder for this preprint (which was not certified by peer review) is the author/funder, who has granted bioRxiv a license to display the preprint in perpetuity. It is made available under a CC-BY 4.0 International license.

Supplementary Information for

Unicellular and Multicellular Modes of Selection Impose Distinct Constraints on Cellular Phenotype Evolution

Mark Kim, Matt Pennell*

*E-mail: mpennell@cornell.edu

1 Simulation

The genotype–phenotype map and evolutionary dynamics are described in the main text under Genotype, regulation, and phenotype and Evolutionary dynamics and simulation.

1.1 Simulation parameters

Table S1 summarizes the parameters used in the main analyses and the values changed in the supplementary analyses.

Table S1. Parameters used in the main and supplementary analyses.

Symbol	Meaning	Main	Supplementary
L	Number of genetic loci	100	—
K	Number of programs	4	6, 8
T	Number of tasks	$2, 4, 6, 8$	$3, 6, 9, 12$ at $K = 6$; $4, 8, 12, 16$ at $K = 8$
m	Number of task-specific phenotypes contributing jointly to fitness	1 to T	$m \in \{1, T\}$ for supplementary robustness analyses
$\overline{\Delta T}$	Mean pairwise separation among task optima	0.2–1.4 in increments of 0.2	same
ρ	Initial density of the genotype matrix	0.25	0.5
γ	Performance-function parameter	1	4
r	Power-mean parameter	0	-2
N	Effective population size	10^4	—
μ	Per-site per-generation mutation rate	10^{-7}	—
n_{rep}	Number of replicate populations per condition	200	—

bioRxiv preprint doi: https://doi.org/10.64898/2026.09.16.751889; this version posted September 17, 2026. The copyright holder for this preprint (which was not certified by peer review) is the author/funder, who has granted bioRxiv a license to display the preprint in perpetuity. It is made available under a CC-BY 4.0 International license.

1.2 Simulation length and termination

The maximum number of substitutions varied with T and m according to

$$S_{\max}(T,m) = \max\left(401,\ \mathrm{round}\left(L\frac{T}{m}\right)+1\right). \tag{S1}$$

When only a small fraction of tasks contributes to fitness during each selective epoch, any particular task is encountered less frequently. We therefore allowed longer simulations at smaller m, with the maximum number of substitutions increasing in proportion to T/m. The substitution limit was at least 401 for every combination of T and m, unless the simulation terminated earlier, allowing comparisons at 50 and 400 substitutions across all conditions. For example, under sequential selection, the maximum is 401 substitutions for $T = 2$ and 4, 601 for $T = 6$, and 801 for $T = 8$. Under simultaneous selection, the maximum is 401 substitutions for every value of T.

Termination also accounted for changes in the active tasks. Under sequential and partially simultaneous selection, the absence of a beneficial mutation for the current task set does not imply that further adaptation is impossible. We therefore drew another set of active tasks whenever no beneficial mutation was available for the current set. Simulations ended when the maximum number of substitutions was reached or when no single-entry mutation was beneficial under any possible combination of m active tasks. We tested the latter condition exactly over all possible combinations of m active tasks. Simulations were also terminated after 100 consecutive task-set draws with no beneficial mutation. For populations that reached a local fitness peak, we retained their final phenotype values in subsequent fixed-substitution comparisons. For populations terminated after 100 consecutive unsuccessful task-set draws, we likewise retained their final recorded phenotype values in these comparisons.

1.3 Task ensembles and replicate populations

Task optima were generated from symmetric Dirichlet distributions as described under Task ensemble construction in the main text. For each combination of T and prescribed $\overline{\Delta T}$, the concentration parameter α was calibrated by bisection in log-space over $\alpha \in [10^{-2}, 10^{2}]$. At each step, 30 task ensembles were generated using the same set of random seeds, and the search continued until their average mean pairwise separation was within 0.02 of the prescribed value, with a maximum of 50 iterations.

Each replicate population was assigned an independently drawn task ensemble at the calibrated value of α and an independently drawn initial genotype. For comparisons among values of m, we used the same initial genotype and task ensemble for the corresponding population in each selection regime. The realized mean pairwise separation among task optima varied across replicate populations, even at the same value of α. To account for this variation, we normalized phenotypic differentiation in each population by the mean pairwise separation among its own task optima. Values of $\overline{\Delta T}$ shown in the figures refer to the prescribed values.

bioRxiv preprint doi: https://doi.org/10.64898/2026.09.16.751889; this version posted September 17, 2026. The copyright holder for this preprint (which was not certified by peer review) is the author/funder, who has granted bioRxiv a license to display the preprint in perpetuity. It is made available under a CC-BY 4.0 International license.

1.4 Summary statistics

Both normalized differentiation and optimization range from zero to one. For a given genome, each phenotype $\mathbf{z}_j(G)$ is the Euclidean projection of its task optimum $\mathbf{T}_j$ onto the convex cone $\{G\mathbf{a} : \mathbf{a} \geq 0\}$. Projection onto this cone cannot increase pairwise distances, so

$$\|\mathbf{z}_i(G) - \mathbf{z}_j(G)\|_2 \leq \|\mathbf{T}_i - \mathbf{T}_j\|_2.$$

Averaging over task pairs therefore gives $0 \leq \overline{\Delta Z}/\overline{\Delta T} \leq 1$.

For optimization, choosing all program coefficients to be zero is always possible, and each task optimum has unit length. The minimum distance to each optimum therefore satisfies

$$0 \leq d_j(G) \leq \|\mathbf{T}_j\|_2 = 1.$$

It follows that $\|\mathbf{d}(G)\|_2 \leq \sqrt{T}$, which gives $0 \leq \Omega \leq 1$.

In Figures 2–4 of the main text and Figures S2–S5, lines show means and shaded bands indicate ± 1 sample standard deviation across 200 replicate populations. The trajectory bands in Figure S1A–C instead show ± 1 standard error of the mean, calculated as the sample standard deviation divided by the square root of the number of contributing populations at each substitution. For Figure S6, we calculated gains relative to sequential selection by subtracting each replicate population's value at $m = 1$ from its value at each m. Corresponding populations shared the same initial genotype and task ensemble across selection regimes. Lines show the mean of these paired differences across 200 populations, and shaded bands indicate ± 1 standard error, calculated as the sample standard deviation of the paired differences divided by $\sqrt{200}$. The gain is zero at $m = 1$ by construction.

1.5 Evolutionary trajectories and comparison points

To set comparison points for earlier and later stages of adaptation, we examined how differentiation, optimization, and the fraction of beneficial mutations changed over successive substitutions (Figure S1). For each substitution, we calculated the fraction of the LK possible single-entry mutations that would increase fitness under the active task set. This fraction was recorded for the task set that produced the substitution (Figure S1C); draws with no beneficial mutation were not included. Under simultaneous selection, populations reached local fitness peaks before 400 substitutions, after which the phenotypic measures no longer changed (Figure S1A–C, see solid lines). Under sequential and intermediate selection, the fraction of beneficial mutations declined rapidly early in adaptation and changed more slowly later, while remaining above zero (Figure S1C, see dotted and dashed lines). The phenotypic measures showed a similar pattern (Figure S1A–B), although differentiation continued to increase under some conditions (Figure S1A). We therefore used 50 and 400 substitutions as heuristic comparison points for earlier and later stages of adaptation, without assuming that all trajectories had strictly stabilized by the later point.

bioRxiv preprint doi: https://doi.org/10.64898/2026.09.16.751889; this version posted September 17, 2026. The copyright holder for this preprint (which was not certified by peer review) is the author/funder, who has granted bioRxiv a license to display the preprint in perpetuity. It is made available under a CC-BY 4.0 International license.

2 Supplementary figures

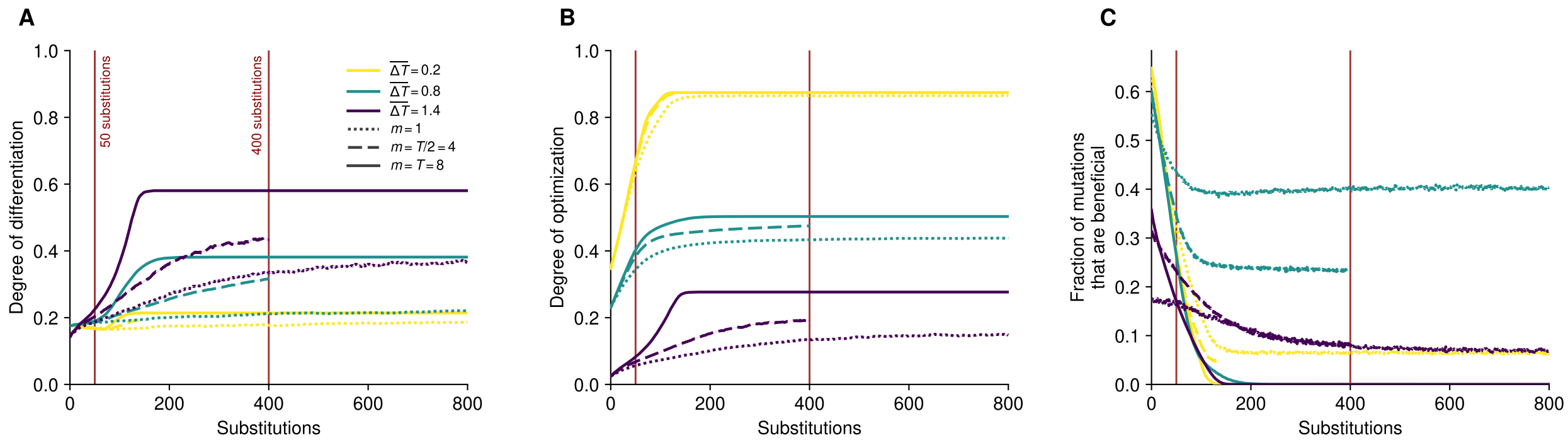

Figure S1. Evolutionary trajectories and the choice of comparison points. A–C. Phenotypic differentiation, optimization, and the fraction of beneficial single-entry mutations over successive substitutions at $T = 8$, with $K = 4$ and $\rho = 0.25$. Colors indicate the prescribed mean pairwise separation among task optima. Dotted, dashed, and solid lines show sequential selection ($m = 1$), selection on half the tasks ($m = 4$), and simultaneous selection ($m = 8$), respectively. Red vertical lines mark the 50- and 400-substitution comparisons. Lines show means and shaded bands indicate ± 1 standard error across contributing populations, from 200 initial replicates per condition. In **C**, the fraction is the number of single-entry mutations that increase fitness under the active task set divided by $LK = 400$. It is recorded for the task set preceding each substitution, excluding task-set draws with no beneficial mutation. Under simultaneous selection, populations reach local fitness peaks where no single-entry mutation can improve fitness. Their final phenotype values are retained thereafter, and their fraction of beneficial mutations is zero. The solid trajectories therefore become flat once all populations have reached these peaks; the flat portions do not represent further substitutions. The $m = 4$ simulations were run for at most 401 substitutions, so the dashed trajectories at the two stronger target separations end near 400. At the weakest separation, the dashed trajectory ends earlier. Trajectories end when fewer than 90% of the original replicates remain available.

Alt text: Three plots show phenotypic differentiation, optimization, and the fraction of beneficial single-entry mutations against the number of substitutions, from zero to eight hundred. Colors indicate separation among task optima, from yellow for weakly separated optima to purple for strongly separated optima. Dotted, dashed, and solid lines show sequential selection, intermediate selection on half the tasks, and simultaneous selection, respectively. Two dark red vertical lines mark the 50- and 400-substitution comparison points, representing the early and late phases of adaptation. In the first two plots, the solid curves rise steeply and then remain flat from roughly 150 substitutions onward, while the dotted and dashed curves continue to change more slowly. In the third plot, the fraction of beneficial single-entry mutations falls steeply from its initial value, reaching zero under simultaneous selection and settling at low positive values under sequential and intermediate selection. Shading shows the standard error across contributing populations.

bioRxiv preprint doi: https://doi.org/10.64898/2026.09.16.751889; this version posted September 17, 2026. The copyright holder for this preprint (which was not certified by peer review) is the author/funder, who has granted bioRxiv a license to display the preprint in perpetuity. It is made available under a CC-BY 4.0 International license.

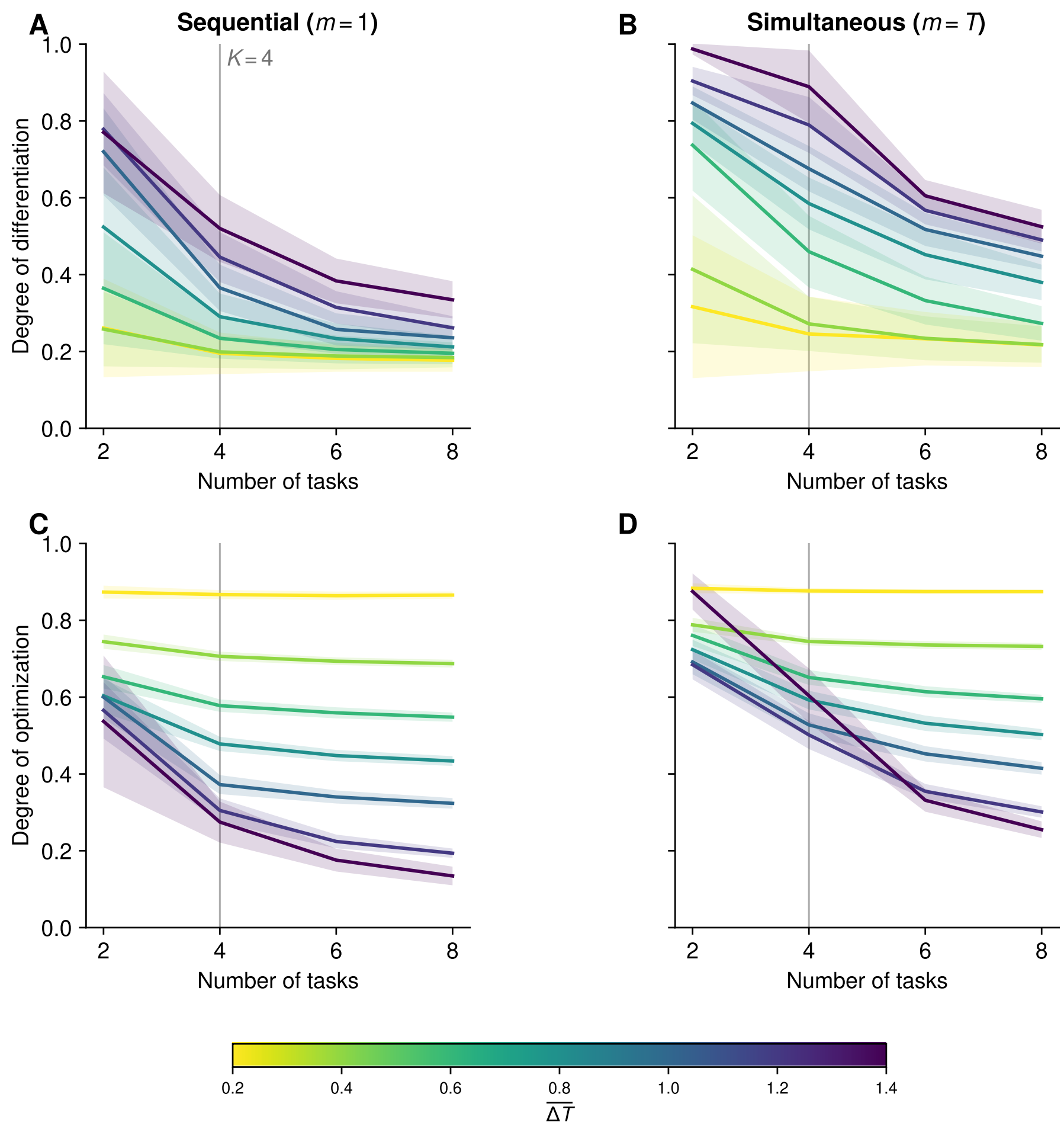

Figure S2. Sequential and simultaneous selection under a negative power-mean fitness function. Fitness is calculated using a power mean with $r = -2$, which gives greater weight to poorly performing tasks. **A, B.** Phenotypic differentiation, $\overline{\Delta Z}/\overline{\Delta T}$, after 400 substitutions under sequential (**A**) and simultaneous (**B**) selection. **C, D.** Optimization index, Ω, under the same conditions. Colors indicate target separation, $\overline{\Delta T}$. Vertical lines mark $T = K = 4$. Under sequential selection, fitness depends on a single task and is therefore independent of r. Panels **A** and **C** repeat Figure 2C,E of the main text for comparison. Lines show means and shaded bands indicate ± 1 standard deviation across 200 replicate populations. Other parameters are unchanged from Figure 2 ($L = 100$, $K = 4$, $\gamma = 1$, $\rho = 0.25$, $\mu = 10^{-7}$, and $N = 10^4$).

Alt text: Four plots compare sequential selection on the left with simultaneous selection on the right, showing differentiation in the top row and optimization in the bottom row against task number. Colors run from yellow for weakly separated task optima to purple for strongly separated optima. Grey vertical lines mark four tasks, equal to the number of programs. Under sequential selection, differentiation falls steeply as tasks accumulate, and the differences among separations narrow. Under simultaneous selection, differentiation remains near its maximum at strongly separated optima while tasks do not outnumber programs, and declines thereafter. Optimization is high and nearly flat for weakly separated optima in both regimes, and declines with task number when optima are strongly separated, most steeply under simultaneous selection. Shading shows one standard deviation across replicate populations.

bioRxiv preprint doi: https://doi.org/10.64898/2026.09.16.751889; this version posted September 17, 2026. The copyright holder for this preprint (which was not certified by peer review) is the author/funder, who has granted bioRxiv a license to display the preprint in perpetuity. It is made available under a CC-BY 4.0 International license.

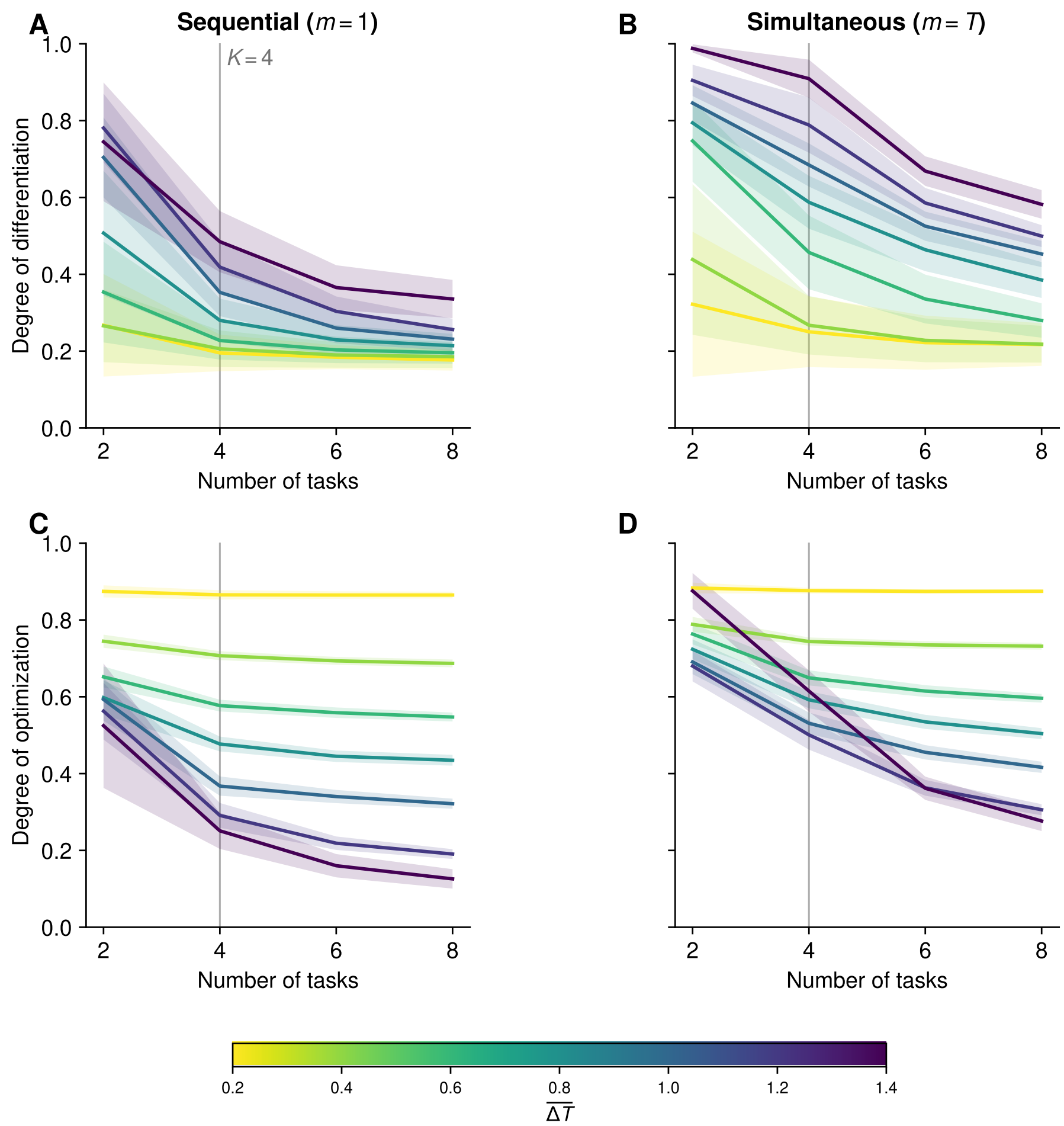

Figure S3. Sequential and simultaneous selection with a more strongly curved performance function. Task performance is calculated using $\gamma = 4$, so performance declines more sharply with distance from the task optimum. Fitness is the geometric mean of performance across active tasks ($r = 0$). **A, B.** Phenotypic differentiation, $\overline{\Delta Z}/\overline{\Delta T}$, after 400 substitutions under sequential (**A**) and simultaneous (**B**) selection. **C, D.** Optimization index, Ω, under the same conditions. Colors indicate target separation, $\overline{\Delta T}$. Vertical lines mark $T = K = 4$. Lines show means and shaded bands indicate ± 1 standard deviation across 200 replicate populations. Other parameters are as in Figure 2 of the main text.

Alt text: Four plots compare sequential selection on the left with simultaneous selection on the right, showing differentiation in the top row and optimization in the bottom row against task number. Colors run from yellow for weakly separated task optima to purple for strongly separated optima. Grey vertical lines mark four tasks, equal to the number of programs. With a more sharply declining performance function, the contrast between the two regimes is unchanged. Under sequential selection, differentiation falls steeply as tasks accumulate, and the differences among separations narrow. Under simultaneous selection, differentiation remains near its maximum at strongly separated optima while tasks do not outnumber programs, and declines thereafter. Optimization is high and nearly flat for weakly separated optima and declines with task number for strongly separated optima, most steeply under simultaneous selection. Shading shows one standard deviation across replicate populations.

bioRxiv preprint doi: https://doi.org/10.64898/2026.09.16.751889; this version posted September 17, 2026. The copyright holder for this preprint (which was not certified by peer review) is the author/funder, who has granted bioRxiv a license to display the preprint in perpetuity. It is made available under a CC-BY 4.0 International license.

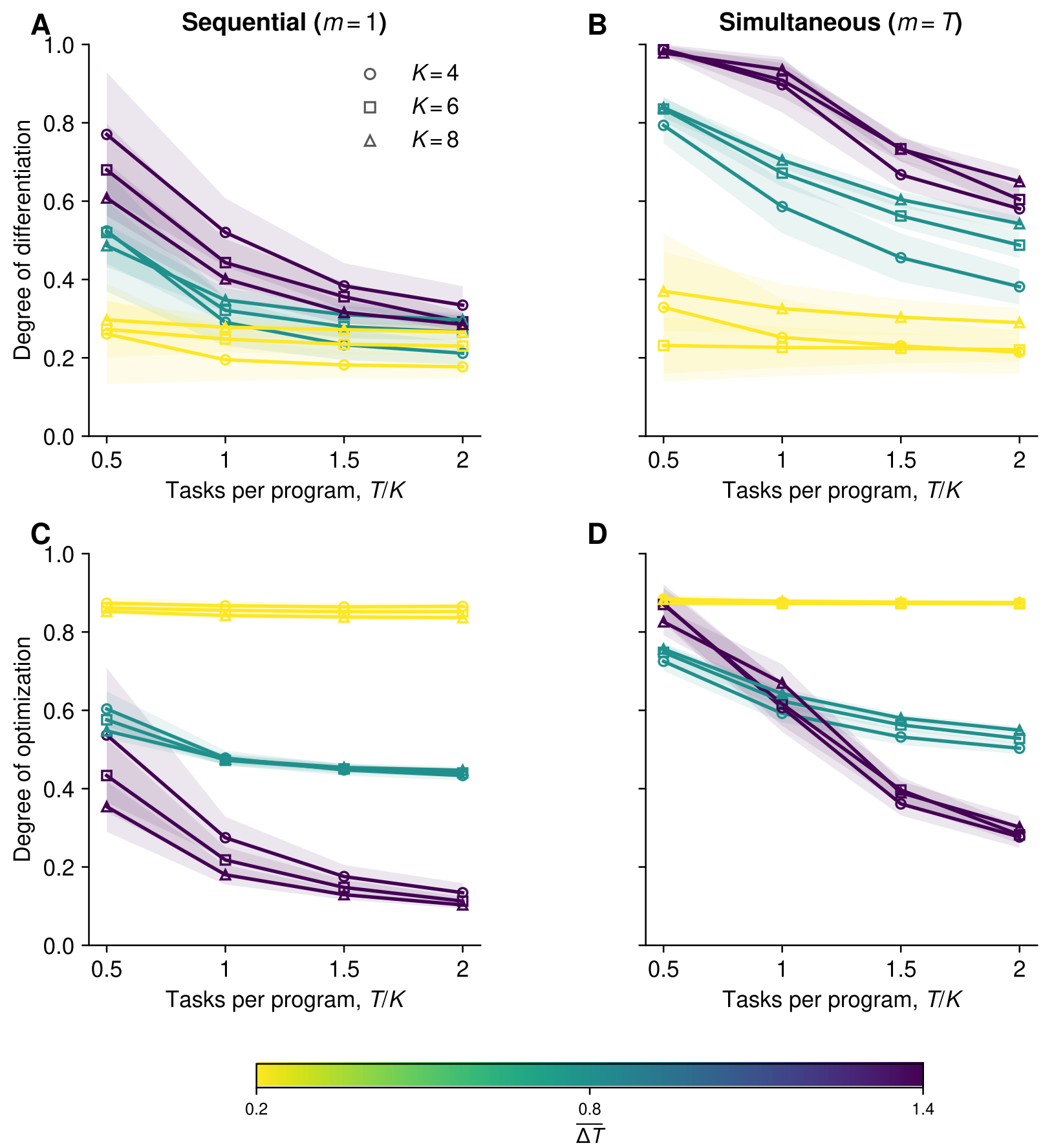

Figure S4. Phenotypic evolution with different numbers of available programs. Sequential and simultaneous selection are compared for $K = 4$, 6, and 8 after 400 substitutions. **A, B.** Phenotypic differentiation, $\overline{\Delta Z}/\overline{\Delta T}$, under sequential (**A**) and simultaneous (**B**) selection. **C, D.** Optimization index, Ω, under the same conditions. Task number is expressed relative to program number, T/K, with equal numbers of tasks and programs at $T/K = 1$. Circles, squares, and triangles indicate $K = 4$, 6, and 8, respectively. Colors indicate target separation, $\overline{\Delta T} = 0.2$, 0.8, and 1.4, as in Figures 2 and 3 of the main text. The initial density is $\rho = 0.25$ for all values of K. Lines show means and shaded bands indicate ± 1 standard deviation across 200 replicate populations. Other parameters are as in Figure 2.

Alt text: Four plots compare sequential selection on the left with simultaneous selection on the right, showing differentiation in the top row and optimization in the bottom row against tasks per program, from one half to two. Circles, squares, and triangles distinguish four, six, and eight programs. Colors indicate weak, intermediate, and strong separation among task optima. Equal numbers of tasks and programs fall at one task per program. Under sequential selection, differentiation declines with increasing tasks per program at every program number. Under simultaneous selection, differentiation remains near its maximum at strongly separated optima up to one task per program, and trajectories for the three program numbers lie close to one another when plotted against tasks per program. Optimization declines with increasing tasks per program in both regimes, most steeply for strongly separated optima under simultaneous selection. Shading shows one standard deviation across replicate populations.

bioRxiv preprint doi: https://doi.org/10.64898/2026.09.16.751889; this version posted September 17, 2026. The copyright holder for this preprint (which was not certified by peer review) is the author/funder, who has granted bioRxiv a license to display the preprint in perpetuity. It is made available under a CC-BY 4.0 International license.

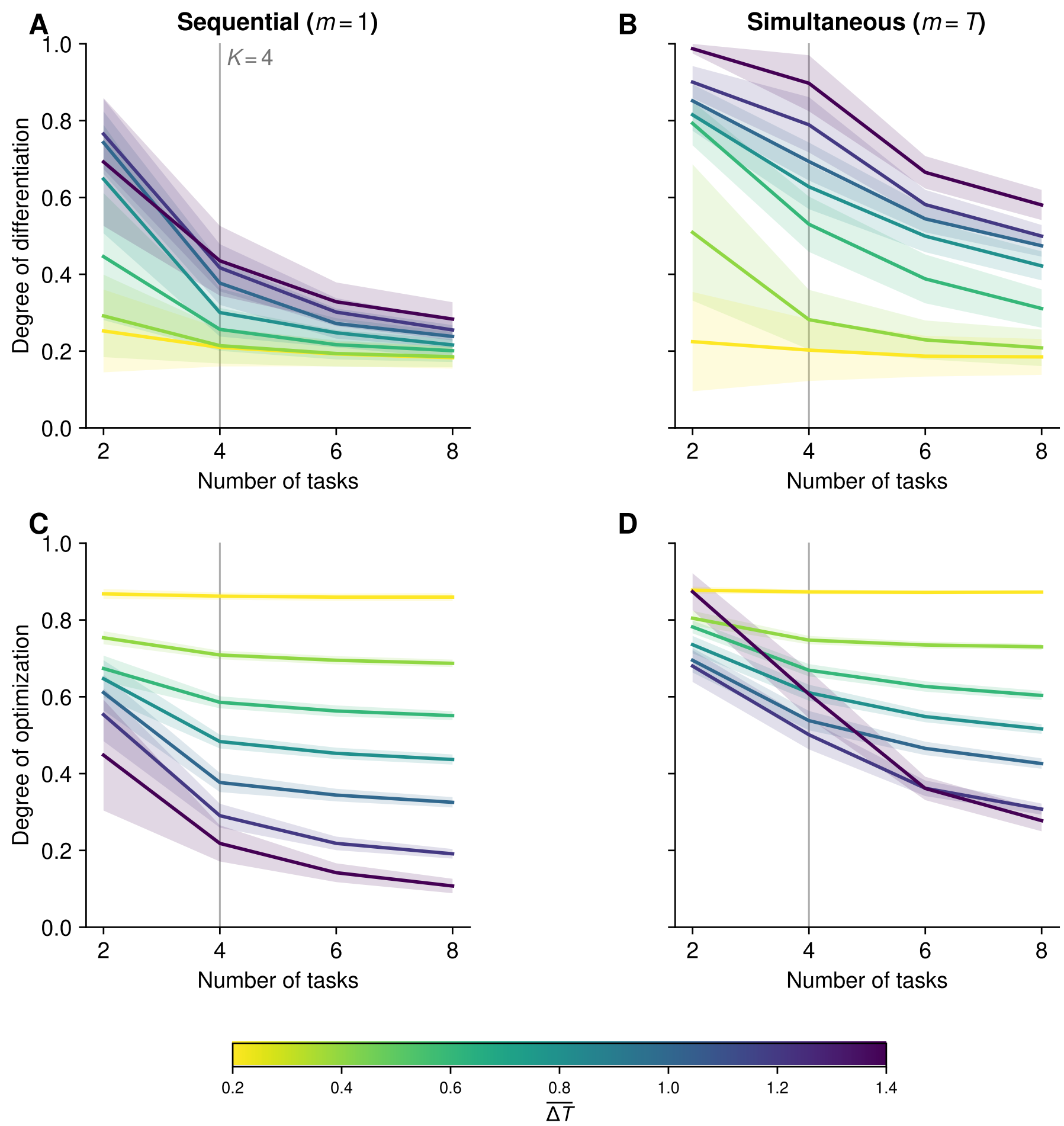

Figure S5. Phenotypic evolution from a denser initial genotype matrix. The initial density is $\rho = 0.5$, so each locus affects two programs on average at $K = 4$, compared with one under the $\rho = 0.25$ initialization. **A, B.** Phenotypic differentiation, $\overline{\Delta Z}/\overline{\Delta T}$, after 400 substitutions under sequential (**A**) and simultaneous (**B**) selection. **C, D.** Optimization index, Ω, under the same conditions. Colors indicate target separation, $\overline{\Delta T}$. Vertical lines mark $T = K = 4$. Lines show means and shaded bands indicate ± 1 standard deviation across 200 replicate populations. Other parameters are as in Figure 2 of the main text.

Alt text: Four plots compare sequential selection on the left with simultaneous selection on the right, showing differentiation in the top row and optimization in the bottom row against task number. Colors run from yellow for weakly separated task optima to purple for strongly separated optima. Grey vertical lines mark four tasks, equal to the number of programs. Starting from a denser genotype matrix does not change the contrast between the regimes. Under sequential selection, differentiation falls steeply as tasks accumulate, and the differences among separations narrow. Under simultaneous selection, differentiation remains near its maximum at strongly separated optima while tasks do not outnumber programs, and declines thereafter. Optimization is high and nearly flat for weakly separated optima and declines with task number for strongly separated optima, most steeply under simultaneous selection. Shading shows one standard deviation across replicate populations.

bioRxiv preprint doi: https://doi.org/10.64898/2026.09.16.751889; this version posted September 17, 2026. The copyright holder for this preprint (which was not certified by peer review) is the author/funder, who has granted bioRxiv a license to display the preprint in perpetuity. It is made available under a CC-BY 4.0 International license.

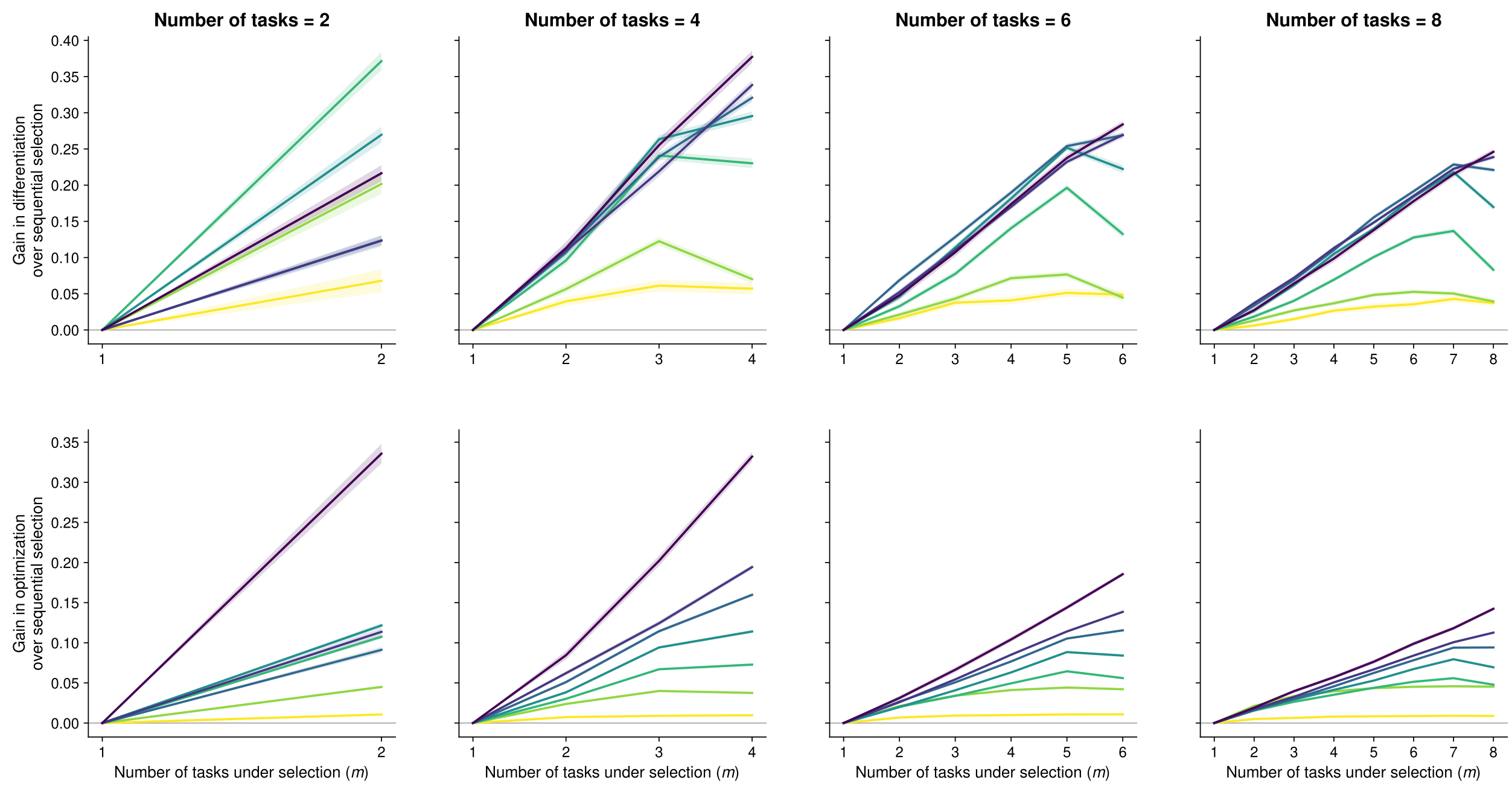

Figure S6. Gains in differentiation and optimization as more phenotypes contribute jointly to fitness. Gains relative to sequential selection are shown against m, the number of task-specific phenotypes contributing jointly to fitness, after 400 substitutions. The top row shows the gain in phenotypic differentiation, $\overline{\Delta Z}/\overline{\Delta T}$, and the bottom row shows the gain in optimization, Ω. Columns indicate task number, T, with $K = 4$ throughout. Colors indicate target separation, $\overline{\Delta T}$. For each replicate, the gain is calculated by subtracting the value at $m = 1$ from that at each m. Gains are therefore zero at $m = 1$, and $m = T$ corresponds to simultaneous selection. At strong target separation, gains are greatest at $m = T$; at weaker separation, they peak at intermediate m. Replicates share the same initial genotype and task ensemble across values of m. Lines show mean paired differences and shaded bands indicate ± 1 standard error across 200 replicate populations. Simulations are the same as those used in Figure 4 of the main text. Parameters are as in Figure 2 and colors follow Figure 2's scale.

Alt text: Eight plots show the gain over sequential selection, with differentiation in the top row and optimization in the bottom row, against the number of task-specific phenotypes contributing jointly to fitness. Columns correspond to two, four, six, and eight tasks. Colors run from yellow for weakly separated task optima to purple for strongly separated optima. Every curve starts at zero, since the gain is measured relative to selection on one phenotype at a time. From four tasks onward, gains in both measures are larger at more strongly separated optima. At strongly separated optima, the gains increase as more phenotypes contribute jointly and are greatest when all of them do. At weaker separations, the curves instead peak before all phenotypes contribute jointly and then decline, an effect that is more pronounced for differentiation than for optimization. Shading shows the standard error of the paired differences across replicate populations.
